# Baby’s first bites: a randomized controlled trial to assess the effects of vegetable-exposure and sensitive feeding on vegetable acceptance, eating behavior and weight gain in infants and toddlers

**DOI:** 10.1186/s12887-019-1627-z

**Published:** 2019-08-01

**Authors:** S. M. C. van der Veek, C. de Graaf, J. H. M. de Vries, G. Jager, C. M. J. L. Vereijken, H. Weenen, N. van Winden, M. S. van Vliet, J. M. Schultink, V. W. T. de Wild, S. Janssen, J. Mesman

**Affiliations:** 1 Institute of Education and Child Studies (trial sponsor), Leiden University, P.O. Box 9555, 2300 RB Leiden, The Netherlands; 2 Division of Human Nutrition and Health, Wageningen University, P.O. Box 17, 6700 AA Wageningen, The Netherlands; 3 Danone Nutricia Research, P.O. Box 80141, 3508 TC Utrecht, The Netherlands; 4 Nutricia Early Life Nutrition, P.O. Box 445, 2700 AK Zoetermeer, The Netherlands

**Keywords:** Complementary feeding, Vegetables, Vegetable exposure, Responsive feeding, Self-regulation of energy intake, Infant, Toddler

## Abstract

**Background:**

The start of complementary feeding in infancy plays an essential role in promoting healthy eating habits. Evidence shows that it is important *what* infants are offered during this first introduction of solid foods: e.g. starting exclusively with vegetables is more successful for vegetable acceptance than starting with fruits. *How* infants are introduced to solid foods also matters: if parents are sensitive and responsive to infant cues during feeding, this may promote self-regulation of energy intake and a healthy weight. However, the effectiveness of the *what* and the *how* of complementary feeding has never been experimentally tested in the same study. In the current project the *what* and *how* (and their combination) are tested in one study to determine their relative importance for fostering vegetable acceptance and self-regulation of energy intake in infants.

**Methods:**

A four-arm randomized controlled trial (Baby’s First Bites (BFB)) was designed for 240 first-time Dutch mothers and their infants, 60 per arm. In this trial, we compare the effectiveness of (a) a vegetable-exposure intervention focusing on the *what* in complementary feeding; (b) a sensitive feeding intervention focusing on the *how* in complementary feeding, (c) a combined intervention focusing on the *what and how* in complementary feeding; (d) an attention-control group. All mothers participate in five sessions spread over the first year of eating solid foods (child age 4–16 months). Primary outcomes are vegetable consumption, vegetable liking and self-regulation of energy intake. Secondary outcomes are child eating behaviors, child anthropometrics and maternal feeding behavior. Outcomes are assessed before, during and directly after the interventions (child age 18 months), and when children are 24 and 36 months old.

**Discussion:**

The outcomes are expected to assess the impact of the interventions and provide new insights into the mechanisms underlying the development of vegetable acceptance, self-regulation and healthy eating patterns in infants and toddlers, as well as the prevention of overweight. The results may be used to improve current dietary advice given to parents of their young children on complementary feeding.

**Trial registration:**

The trial was retrospectively registered during inclusion of participants at the Netherlands National Trial Register (identifier NTR6572) and at ClinicalTrials.gov (NCT03348176).

Protocol issue date: 1 April 2018; version number 1.

## Background

In light of today’s global obesity epidemic and related diseases, promoting healthy eating habits is essential [1]. Children as young as 1–3 years of age already eat too much energy-dense food and too little fruit and vegetables [2–6]. In the Netherlands, based on surveys between 2006 and 2014, estimates for the percentage of preschoolers failing to meet daily recommendations for vegetable intake vary from 40% up to an alarming 80% [2, 3]. Moreover, a recent experimental study showed that almost 40% of 4 year-olds fail to effectively regulate their own energy intake, showing a tendency to eat even though they are not hungry [7]. Poor eating habits, such as consuming too little vegetables and eating in the absence of hunger increase the risk of developing overweight and obesity, and related diseases such as type II diabetes [8–12], cardiovascular disease [13], and certain cancers [14]. Both children’s food preferences and their ability to self-regulate their energy intake are influenced by their direct environment already in the first two years of life [15–20]. Therefore, promoting a healthy diet and healthy eating habits and behavior from infancy is essential. At this young age, parents bear primary responsibility for the diet of their children. The present article describes the study protocol and sample of a randomized controlled trial under the acronym *Baby’s First Bites (BFB),* aimed at (a) promoting vegetable intake and liking, and (b) promoting child self-regulation of energy intake, by advising parents *what* and *how* to feed their infants from the very start of complementary feeding. The primary goals of promoting vegetable acceptance and self-regulation of energy intake serve the purpose of reducing the risk of developing overweight in early childhood – our secondary outcome. Three interventions will be compared to an attention-control condition (1): a *repeated exposure* intervention motivating parents to repeatedly expose their children to the taste of a variety of vegetables during the first year of complementary feeding (2); a *parenting* intervention promoting sensitive parental feeding; and (3) a *combined* intervention promoting both repeated exposure to vegetables and sensitive feeding.

### Repeated exposure to a variety of vegetables from the start of complementary feeding

When parents start complementary feeding, they can choose from a variety of foods to introduce to their children, including (baby) cereals, grains, fruits or vegetables [21, 22]. Already in the 1970s it was theorized that to improve the acceptance of vegetables, these should be introduced before fruits or other sweet tastes during complementary feeding because infants’ inherent preference for sweet tastes will interfere with vegetable acceptance [23]. The effects of starting complementary feeding exclusively with vegetables on promoting vegetable acceptance has, however, not been studied often [24]. Two other methods of increasing vegetable intake and liking *have* been studied extensively. First, repeated exposure to the taste of vegetables has been shown effective in increasing its intake and liking in infants and preschoolers [24–32], especially for bitter tastes [33]. Second, being exposed to a variety of vegetables increases vegetable acceptance in infants [23, 29, 34, 35]. However, whether it is indeed most effective to start with vegetables *only* was not tested until the trial by Barends et al. in 2013 [22]. This study showed that infants exposed to a variety of vegetables during the first three weeks of complementary feeding – including a target vegetable to which they were repeatedly exposed – nearly doubled their intake of the target vegetable, whereas children who only received fruits showed increased intake of fruits but not of vegetables [26]. Shortly after this trial, another intervention study found similar results: encouraging parents from the United Kingdom to start complementary feeding with a variety of vegetables significantly increased vegetable intake compared to a control group in which parents were allowed to start complementary feeding with whatever food they wanted [36].

Thus, there is preliminary evidence that starting complementary feeding by repeatedly exposing infants to a variety of vegetables is an effective way to increase vegetable intake and liking in the first year of a child’s life. However, the beneficial effects on vegetable acceptance do not seem to last when children grow older [27, 30, 37]. This is in line with the finding that children are open to trying a variety of different tastes in their first year of life, but tend to become more selective about their diet when they become older (especially in the ‘food neophobic phase’) [24, 38, 39]. Indeed, in the Barends et al. trial, starting complementary feeding with vegetables did *not* predict vegetable intake at age two, whereas how selective children were about what they wanted to eat did [27]. Continuing the active promotion of eating vegetables in the first and second year of the child’s life after exposing them to a variety of vegetables at the start of complementary feeding may counteract the negative effects of the food neophobic phase and effectively boost vegetable intake throughout childhood. However, most intervention studies have been conducted with infants in the early phases of complementary feeding or preschoolers older than 2 years; few studies focus on promoting vegetable acceptance in the difficult period between 12 and 24 months when children go through the major transition of eating the same meals as their family and enter the food neophobic phase [40, 41]. Therefore, we studied the effectiveness of a more prolonged vegetable-exposure intervention throughout the whole first year of complementary feeding, well into the more ‘difficult’ second year of the child’s life to promote vegetable intake in toddlers.

### Sensitive feeding

Apart from *what* parents should offer their children during complementary feeding, *how* they offer this food may also strongly influence a child’s acceptance of the offered food, as well as their ability to self-regulate their energy intake. Experimental studies show that pressuring a child to eat decreases children’s ability to self-regulate their energy intake and thereby to consume appropriate amounts of calories [42]. Similarly, pressuring a child to eat vegetables has a counterproductive effect and will make a child eat and like these vegetables less [43]. Even giving subtle prompts to eat, like moving food towards a child, may have a counterproductive effect [44]. However, if children start to decrease their vegetable intake when they enter the second year of life, parents are likely to use some sort of pressure to make their child eat. Indeed, an Australian study showed that more than half of the parents of 1–3 year-olds sometimes insist on their child eating a food, and 35% reported to pressure their child often or all the time [45]. As such, it is not surprising that many parents struggle with the question how to feed their infants effectively. Indeed, 25 to 40% report feeding problems with their infants and toddlers, including picky eating and strong food preferences [46, 47].

In contrast to pressuring children to eat, *responsive feeding* is often suggested to be the optimal way to feed infants and toddlers [48–51]. Responsive feeding is generally defined as a style of feeding in which parents correctly perceive the hunger and satiety signals of the child, and respond promptly and appropriately [50, 52]. This feeding style is suggested to promote and reinforce young children’s ability to self-regulate their energy intake, because the parent who feeds responsively will not override a child’s satiety cues [50]. Indeed, promoting responsive feeding was shown to be associated with a reduced risk of overweight and of rapid weight gain during the first years of life [50, 53, 54]. However, although attending to hunger and satiety signals may promote child self-regulation of energy intake, it may not be sufficient to promote healthy food preferences including vegetable acceptance during the first years of the child’s life. As children from the age of 1.5 years become more and more autonomous and selective about their food preferences, parents have to manage that their child eats appropriate quantities, but also the specific (healthy) foods that are served. To promote healthy food preferences, parents will need to stimulate their child to eat vegetables in a non-pressuring way that is sensitive to the child’s autonomy-related needs and behaviors. This requires more than just responsiveness to hunger and satiety cues, but also sensitive discipline strategies to challenging child behavior (e.g. when a child throws their food on the ground) and sensitive responses to distracted behavior (e.g. when a child is more interested in what is happening around them than in its plate of food). Sensitive discipline strategies that parents may use entail positive encouragement (e.g. explicitly complimenting the child for positive behavior), appropriate pacing to allow the child sufficient time to adapt to the situation, granting the child appropriate amounts of autonomy (e.g. allowing the child to eat autonomously when the child is able to and shows it wants to) and showing understanding for the child’s point of view [55]. Using these sensitive discipline strategies has been shown to promote infant’s committed compliance, i.e. internally motivated and self-regulated adherence to parental rules [56]. In the current study we introduce the concept *sensitive* feeding to capture this broader set of sensitive parenting skills relevant to promoting children’s committed compliance to parental attempts to feed them healthy foods. Sensitive feeding thus includes the traditional concept of responsive feeding [50, 52], but with the addition of sensitive discipline as well as autonomy support, also in response to non-food related child behaviors during feeding. We hypothesize that parents showing sensitive feeding will be more successful in increasing their children’s vegetable acceptance.

In recent years a number of randomized controlled trials to promote responsive feeding have been performed, some of which incorporated the discipline component described above [57–62] whereas others merely focused on teaching parents how to effectively respond to the hunger and satiety cues of their child [53, 54]. However, none of these interventions focused on promoting responsive or sensitive feeding alone. Instead, they incorporated a much broader range of topics such as dietary advice, advice on general feeding practices, guidelines for physical activity, or even more broad advice on how to manage the sleeping and crying behavior of the child. As such, it is impossible to isolate the specific effect of responsive feeding on the diet and eating behavior of the child, and whether this is in fact an element that should be targeted to promote healthy eating patterns. Moreover, all previous trials evaluated changes in parenting behavior via self-report questionnaires, whereas expert observations of parent-child interaction is considered the gold standard to measure parenting behavior [63]. An important disadvantage of self-reports of parenting behavior specifically is that it is questionable whether these data represent the actual parenting behavior parents show, or rather attitudes about what they think they are or should be doing. Indeed, the correlation between self-reported and observed parenting behavior is often low, both in the field of parental feeding [64–66] and in other fields [67]. Therefore, we will test the effectiveness of an intervention focusing solely on the enhancement of sensitive feeding, by evaluating its outcomes using repeated observations of family meals at home in addition to self-reports.

### Repeated exposure and sensitive feeding

Whether a combination of repeatedly exposing infants to vegetables and encouraging sensitive feeding may lead to a better vegetable intake and liking than each of the interventions alone, has never been tested before. However, there is evidence that presentation of beneficial food choices (succeeding at the *what*) in a non-responsive manner (failing at the *how*), and the presentation of unhealthy food choices (failing at the *what*) in a responsive manner (succeeding at the *how*) may lead to overweight and eating problems in children [43, 68]. For instance, an experimental study by Galloway and colleagues showed that pressuring a child to eat, even if this pressure is mild in nature, decreases the beneficial effects of repeated exposure to the taste of vegetables [43]. This suggests that an intervention aimed at both elements may be particularly powerful.

### Aims and hypotheses

In summary, the *Baby’s First Bites (BFB)* study aims to test whether promoting the *what* and**/**or promoting the *how* of complementary feeding will result in increased vegetable consumption and liking and a better self-regulation of energy intake in infants and toddlers up until the age of 36 months. To this end, we will perform a superiority randomized controlled trial with parallel groups, comparing a) an intervention focusing on vegetable exposure (=what), b) an intervention focusing on sensitive feeding (=how), c) an intervention focusing on vegetable exposure and sensitive feeding (=what and how), and d) a control condition. The interventions will begin when the infant starts receiving complementary food (child age 4–6 months, as recommended by the Dutch Nutrition Center) and continue until the child is 16 months old. We hypothesize that a) all interventions are more effective in improving vegetable consumption and vegetable liking than the control condition without guidance on complementary feeding; b) the sensitive feeding and combined intervention will be more effective in supporting child self-regulation of energy intake than the vegetable exposure or control conditions; and c) the combined intervention is more effective than the other two interventions alone in promoting vegetable intake and vegetable liking. As the inclusion phase of the *BFB* study has already successfully been completed, the present article describes the characteristics of the sample of included participants as well as the design of this ongoing study.

## Methods/design

### Study design

**Fig. 1:**
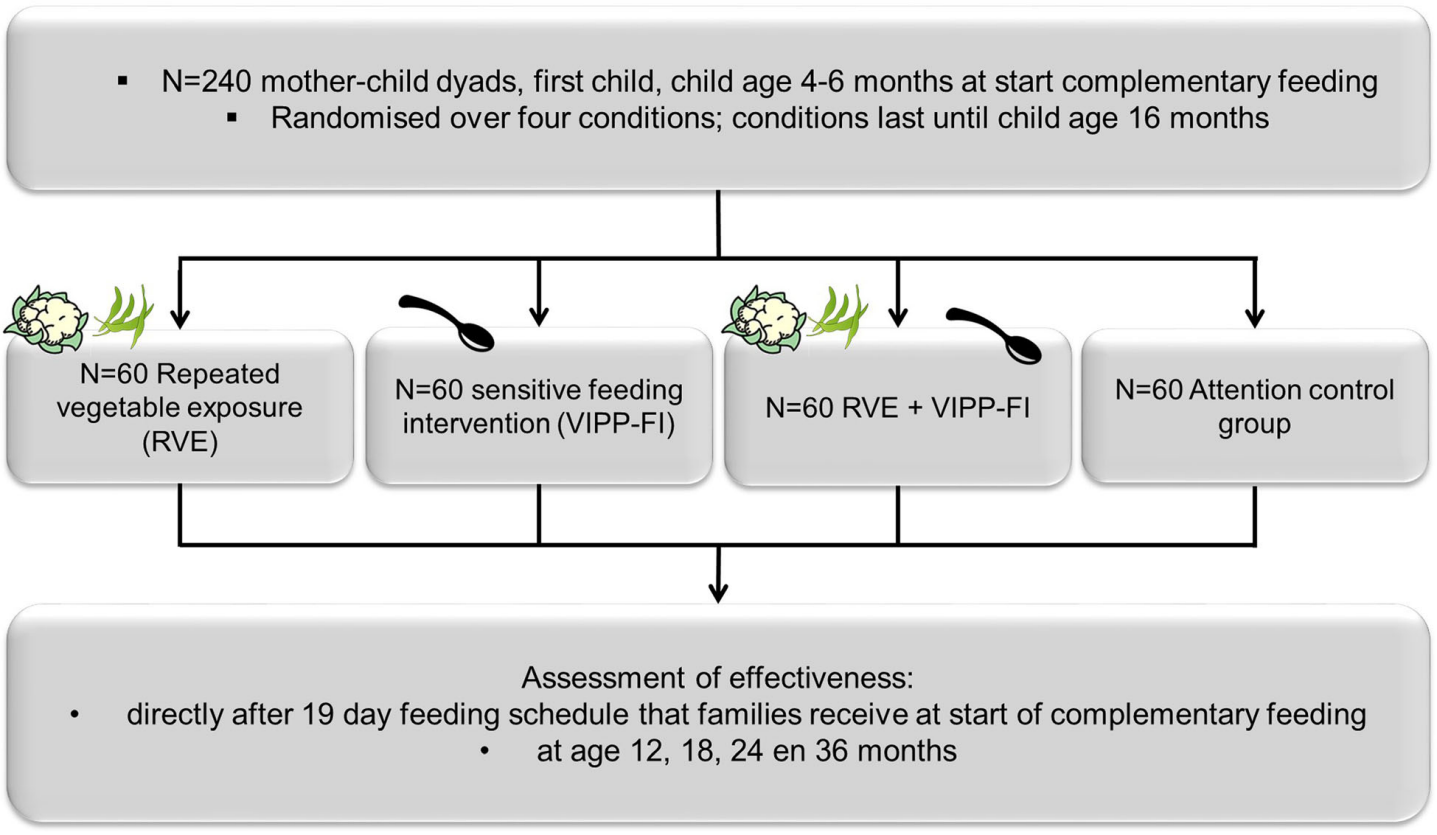
General overview of study design

The *BFB* study is a collaboration between Leiden University, Wageningen University and Research, Danone Nutricia Research and Nutricia Early Life Nutrition. The study is a multicenter trial that is currently being performed at Leiden University and Wageningen University and Research, using a superiority randomized controlled design. The protocol has been approved by the Ethical Review Board of Education and Child Studies, Leiden University (protocol number ECPW-2015/116) and the Medical Ethical Review Board of Wageningen University and Research (METC-WU protocol number NL54422.081.15). The inclusion phase started in May 2016 and ended successfully in November 2017. Mothers and their 4–6 month-old infant were randomly allocated to receive either repeated exposure to a variety of vegetables (RVE), the parenting intervention Video-feedback Intervention to promote Positive Parenting-Feeding Infants (VIPP-FI), RVE and VIPP-FI combined, or an attention-control intervention (see Fig.1 and Table 1). Families receiving the RVE intervention were further randomly allocated to one of two types of vegetables the infant is repeatedly exposed to (see *Interventions* below): green beans or cauliflower. Two target vegetables were chosen as the current feeding schedule is based on the 19-day feeding schedule as described by Barends and colleagues [26, 27]. Green beans and cauliflower are commonly consumed in the Netherlands. Randomization into these conditions was done using the online program TenALEA, which assured that the exact same randomization procedure was used at both study sites [69]. To make the groups allocated to the different conditions as comparable as possible concerning relevant potential confounders, randomization was stratified by age of the child at the start of complementary feeding (4, 5 or 6 months), gender of the child and study location, using minimization procedures. The online randomization program TenALEA has been used previously in other clinical trials [70, 71]). Participants were allocated to a condition by one of the PhD-students or research assistants at each study location.

**Table 1:** Overview of conditions and intended N per condition

Name	Description of condition	N
RVE	Repeated vegetable exposure intervention: - exposure to either green beans or cauliflower as target vegetable during the first 19 days of weaning - five phone calls to motivate parents to expose children to vegetables at child age 4–6, 8, 13 and 16 months	60
VIPP-FI	VIPP-Feeding Infants: - exposure to fruits and a sweet vegetable (carrots) during the first 19 days of weaning - five home-visits using video-feedback to promote sensitive feeding at child age 4–6, 8, 13 and 16 months	60
COMBI	Combination of RVE and VIPP-FI	60
AC	Attention control group: - exposure to fruits and a sweet vegetable (carrots) during the first 19 days of weaning - five phone calls on development of child at age 4–6, 8, 13 and 16 months	60

*Note*. *RE* Repeated exposure, *VIPP-FI* VIPP-Feeding infants, *COMBI* Repeated exposure and VIPP-Feeding infants combined, *AC* Attention-control condition

Intervention effects are assessed both during and after conclusion of the interventions by performing a pre-test at the first two days of complementary feeding (child age 4–6 months; *t*_*0*_), two assessments during the interventions (at the end of the 19-day feeding schedule (child age 5–7 months; *t*_*1*_) and when the child is 12 months old (*t*_*12*_)), a post-test at the age of 18 months (*t*_*18*_) and two follow-ups when the child is 24 (*t*_*24*_) and 36 months old (*t*_*36*_). *T*_*0*_ and *t*_*1*_ are not scheduled at a standard, fixed child age but rather within a certain age range because we wanted to allow parents to start complementary feeding when they thought their child was ready. The other measurements *are* scheduled at set child ages because the intervention sessions following the very first start of complementary feeding are scheduled at fixed time points (see *Timing of intervention sessions* below). The timeline for participants is depicted in Table 2. Participants are allowed to stop at any point during the study if they no longer want to participate. If participants decide to withdraw from the study, discontinue an intervention or are unable to complete a specific assessment, they will be asked once whether they would still be willing to complete (parts of) the intervention, the post-test and/or follow-up assessments to come.

**Table 2:** Timeline for participants

	Enrolment	Intervention-period						Post-test	Follow-up	
Child age (in months)	2–4	4–7		8	12	13	16	18	24	36
Time point		*t*_*0*_	*t*_*1*_		*t*_*12*_			*t*_*18*_	*t*_*24*_	*t*_*36*_
Enrolment & allocation										
1. Invitation e-mail	x									
2. Information and informed consent	x									
3. Screening	x									
4. Allocation	x									
5. Rice-flour porridge	x									
Interventions										
RVE										
Feeding schedule		days 1–19								
Phone-call		Twice in period days 3–17		x		x	x			
Provision of vegetable purees		X		x	x					
VIPP-FI										
Feeding schedule		days 1–19								
Home-visit		Twice in period days 3–17		x		x	x			
Provision of fruit and carrot purees		X		x	x					
Combined RVE + VIPP-FI										
Feeding schedule		days 1–19								
Phone-call + home-visit		Twice in period days 3–17		x		x	x			
Provision of vegetable purees		X		x	x					
Attention-control										
Feeding schedule		days 1–19								
Phone-call		Twice in period days 3–17		x		x	x			
Provision of fruit and carrot purees		X		x	x					
Assessment of study outcomes^a^		Days 1 + 2	Days 18 + 19		x			x	x	x

*Note.* ^a^Primary outcomes: vegetable intake and liking, child self-regulation of energy intake. Secondary outcomes: child anthropometrics, child eating behavior and maternal feeding behavior. RVE = repeated exposure to vegetables. VIPP-FI=Video-feedback Intervention to promote Positive Parenting-Feeding Infants

### Calculation of sample size

A power analysis was conducted to calculate the sample size necessary to detect a moderate effect size of .50, which is based on previous studies of the effects of repeated exposure to vegetables [27] and the effects of VIPP [72]. Given a power of .80 and an alpha of .05 the analysis showed that a sample size of 51 participants per group would be sufficient. Taking attrition into account, we aimed to include a total of 240 mothers, 60 per group (see Fig. 1 and Table 1).

### Recruitment and participants

**Fig. 2:**
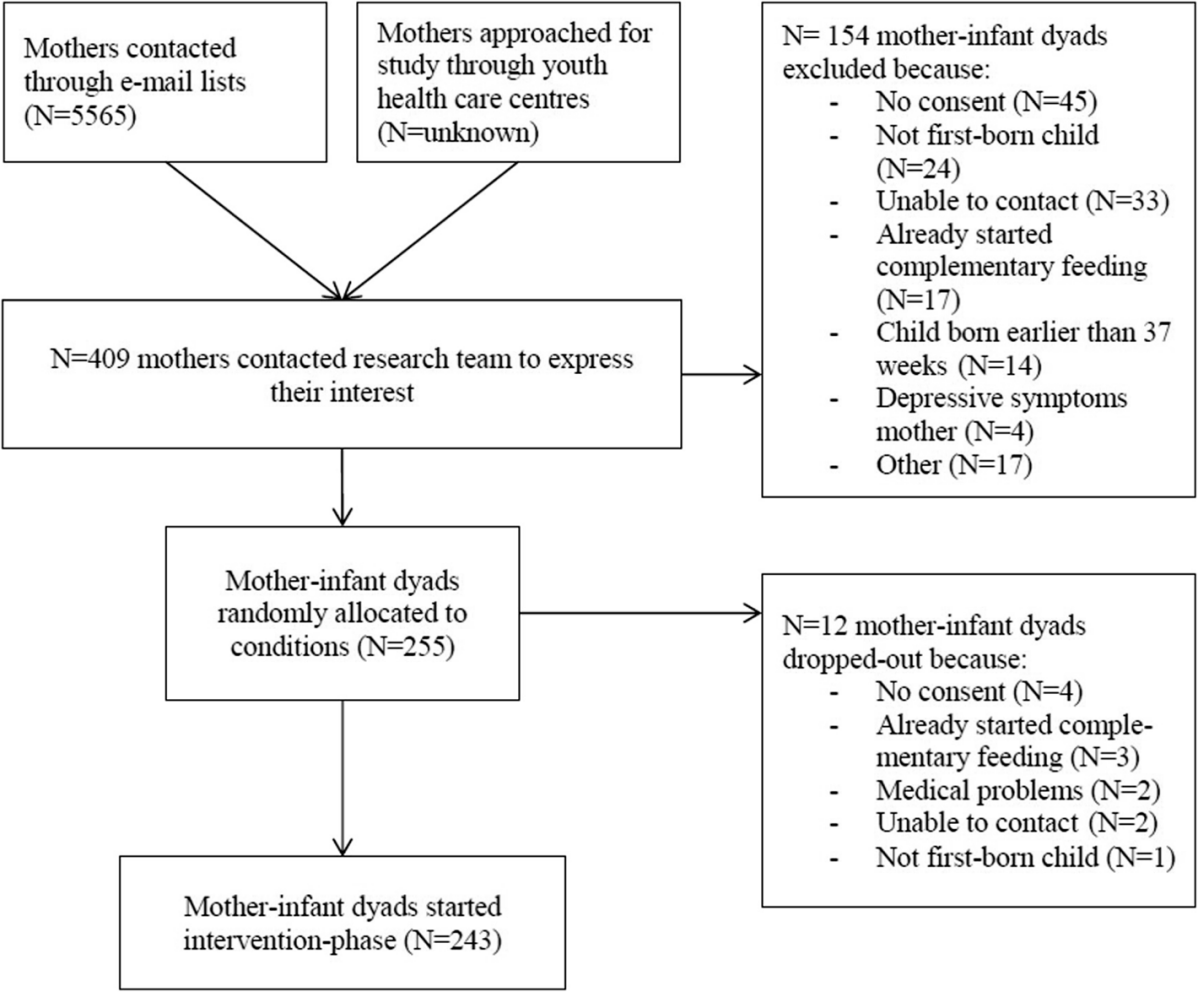
Flowchart of the inclusion phase

We decided to focus all interventions on mothers, because in Dutch households women most often fulfil the role of primary caregiver. Participants were recruited from the general population in four Dutch provinces (Zuid-Holland, Noord-Holland, Gelderland and Utrecht) that are closest to the two universities performing the trial, Leiden University and Wageningen University and Research. Participants were recruited by sending emails with information about the study and a link to the website of the study to mothers of 2–4 month-old infants. Addressees included parents who had signed up for the ‘Nutricia for parents group’ or were parents who had ordered a free gift box containing baby merchandise from ‘WIJ Special Media’. All addressees had indicated that they were interested in receiving information on additional opportunities and/or activities. Names and e-mail addresses were available to only a limited number of researchers, ensuring the privacy of the addressees. Finally, we approached potential participants through handing out brochures at youth health care centers within the vicinity of Wageningen University and Research. We cannot ascertain how many families were invited at the youth health care centers, but the total number of families invited through the two e-mail lists was 5565. A total of 409 families expressed interest in our study, 255 of which fulfilled in- and exclusion criteria (see below) and were randomly allocated to the groups (62.3%; see Fig. 2).

Families that showed interest in our study received a phone-call from one of our trained researchers/students, explaining the study in detail. Families still expressing interest in the study at the end of the call received a detailed information brochure as well as consent forms. Both mothers and fathers were asked to sign and return the consent forms. After receiving the signed consent forms, mothers were asked to fill out an online screening questionnaire which assessed inclusion criteria. Families had to fulfil the following inclusion criteria: a) first-time mothers; b) healthy term infants (37–42 weeks of gestation); c) planning to start complementary feeding at child age of 4–6 months (families that already started complementary feeding were excluded) and d) sufficient knowledge of the Dutch language to receive advice on complementary feeding in Dutch and to be able to fill out Dutch questionnaires. Mothers with major psychiatric diagnoses (e.g., depression, schizophrenia or borderline personality disorder) were excluded, as these may affect parenting [73]. Following the study protocol of Barends and colleagues [26], families were also excluded when the first-borns were twins or in the case of medical problems in the infants that influence the ability to eat, such as food allergies, swallowing or digestion problems. Finally, for standardization purposes, mothers who were not willing to commit to the outcome of the randomization procedure were excluded, e.g. the child was assigned to a VIPP-FI group, but the mother was objecting to being video-taped. A flowchart of the inclusion phase can be found in Fig. 2.

In total, 255 first-time mothers and their babies were randomly allocated to the various conditions. Directly after randomization, prior to starting the intervention-phase, 12 mother-infant dyads dropped out (for reasons, see Fig. 2). A total of 243 families successfully started the intervention-phase. Mean age of the mothers was 30.4 years (*sd* = 4.7, range 18–44). Concerning educational level, 41.6% of mothers had a lower education (finished high school or vocational school), 38.7% finished higher education (higher vocational school) and 19.8% finished university. The trial was thus successful in including a large group with lower education, which is generally considered a risk factor for having less healthy eating habits [74] and less beneficial parental feeding styles [75]. About 18% of mothers worked fulltime, and 63 worked part-time, and 19% did not have paid work Gender of the child was roughly equally distributed (47.3% boys); mean age of the children at the start of the intervention-phase was 4.68 months (*sd* = .42, range 3.98–6.38 months); median age was 4.57 months.

### Interventions

The specific content and timing of the RVE and VIPP-FI interventions are specified in Table 1. To control for possible placebo-effects due to receiving attention from researchers/interveners, the number of contacts with researchers/interveners and time in between contacts are the same for all conditions. The interventions in all groups as well as the attention control condition is performed by trained researchers or Master’s students in the fields of nutrition or child and family studies. Participants in all conditions are allowed to seek any type of concomitant advice on infant feeding during the trial; to control for potential co-intervention bias we ask participants after the interventions are completed whether they sought advice concerning feeding elsewhere, and if so, where and how often.

## All groups/conditions

### Feeding schedule and provision of foods in all groups

Prior to the start of each intervention, all mothers are instructed to give their infant rice-flour porridge with a spoon for at least five days, to accustom the infant to eating food from a spoon [26]. Each intervention starts with providing infants their first bites of complementary foods according to a specific 19-day feeding schedule (see Table 3). The infants in the repeated exposure and combined conditions receive a variety of commercially available jars of vegetable purees, whereas the infants in the VIPP-FI and attention-control condition receive similar jars containing both fruits and a sweet vegetable puree (carrots). During the first two days and the last two days of the feeding schedule, the target and control vegetables (cauliflower and green beans) are provided to infants in all conditions. During these days, families are visited at home by the research team and the feed is videotaped; researchers measure at home how much the child has eaten (see *Measures*). During the other days of the feeding schedule, the mother feeds her child at home without the presence of the researchers. To facilitate compliance to the feeding schedule, mothers receive a printed overview of the feeding schedule indicating which puree to feed their child on each of the 19 days. In addition, each jar of food is labelled with a sticker indicating the day of the feeding schedule.

**Table 3:** Feeding schedules used within each intervention group and the control group

	Day																		
Condition	1	2	3	4	5	6	7	8	9	10	11	12	13	14	15	16	17	18	19
RE and COMBI	TV	CV	TV	V1	TV	V2	TV	V1	TV	V2	TV	V1	TV	V2	TV	V1	TV	CV	TV
VIPP-FI and AC	CF	GB	F1	F2	F3	V3	F1	F2	F3	V3	F1	F2	F3	V3	F1	F2	F3	GB	CF

*Note*. *RE* Repeated exposure, *COMBI* Repeated exposure and VIPP-Feeding infants combined, *VIPP-FI* VIPP-Feeding infants, *AC* Attention-control, *TV* Target vegetable (either green beans or cauliflower), *CV* Control vegetable (either green beans or cauliflower); *V1* Spinach, *V2* Broccoli, *CF* Cauliflower, *GB* Green beans, *F1* Apple, *F2* Pear, *F3* Banana; *V3* Carrot

After this feeding schedule has been completed, all families are provided with a total of 100 jars of age-appropriate fruits and/or meals with vegetables, depending on the condition they are in, up until the child is approximately 12 months of age (distributed on five different occasions; 20 jars per occasion). Parents are free to decide whether they want to feed their baby using homemade foods or the jars provided to them. The provision of these foods serves as a means to facilitate prolonged exposure to vegetables in the repeated vegetable exposure conditions by making sure age-appropriate meals containing vegetables are available to the families. Whether or not families use these jars and how much the child eats of these jars is reported by the mother.

### Timing of intervention sessions

The five sessions of each intervention and the phone calls in the control condition are timed to take place when the infant goes through major transitions in eating (see Table 2). It was decided to give advice specifically during these major transitions to optimize the potential effectiveness of the interventions. The first two sessions are scheduled when the infant has just started eating complementary foods (approximately one and two weeks after the start). The third session is scheduled when the child reaches the age of 8 months and parents should start introducing their child to more lumpy foods to facilitate their infants’ acceptance of different food textures [76]. The fourth session is scheduled when the child is approximately 13 months and is allowed to eat the same foods as the rest of the family. Finally, the fifth session is scheduled when the child is 16 months of age to prepare parents for the potentially difficult ‘food neophobic phase’ that infants tend to reach in their second year [39, 77].

## Repeated exposure to a variation of vegetables (RVE)

The repeated vegetable exposure (RVE) intervention focuses on *what* to feed infants. The RVE intervention starts with vegetables only according to a 19-day feeding schedule as described by Barends and colleagues [26, 27], and further promotes vegetable exposure in the first year of complementary feeding until 16 months of age using a protocol developed specifically for the current study. We conducted a needs assessment and applied the Intervention Mapping (IM) process [78–80] to develop this protocol.

In short, to promote vegetable exposure in the first year of eating complementary foods the method of repeated exposure to vegetables is used because it has been found to be the most effective way to increase vegetable intake and liking in infants [40, 81]. To support this method, we motivate mothers both during and after the feeding schedule to offer their child vegetables daily. From an analysis of risk factors and determinants that may influence children’s vegetable consumption we selected the determinants *knowledge, attitude, self-efficacy, skills, modelling, availability of vegetables, beliefs of the parent, positive reinforcement,* and *costs* to target in the intervention.

The main goal of the RVE intervention is for mothers to increase the child’s acceptance and liking of vegetables by a) starting the first 19 days of complementary feeding with vegetables only and b) offer vegetables to their child daily after this first period. The risk factors and determinants described above are targeted with the feeding schedule and the five telephone calls. Each phone call focuses on a different theme (Table 4) and discusses basic information material and optional additional information material that is sent to mothers by post. Mothers are asked to read the basic information before the scheduled telephone call with the researcher. Conversations are structured according to the general principles of Motivational Interviewing (MI) [82]. Interveners are instructed to act as a coach and guide mothers through the feeding schedule and – during later sessions – the family meal. The telephone protocol contains guidelines with questions mothers might ask and possible responses.

**Table 4:** Content of each of the RVE and VIPP-FI intervention sessions

Session	Child age	RVE			VIPP-FI	
		Theme	Topics discussed	Optional information	Situation filmed	Topics discussed
1	4–6 m	Discovering vegetables	• Why should children learn to eat vegetables? • Keep offering, also if child rejects (at least 10 times)	• Benefits of eating vegetables • Development of taste in young children	Mother feeding infant pureed vegetables/fruits	Learn to observe and interpret child feeding cues (hunger, satiation, liking)
2	4–6 m	Keep on offering vegetables	• How long should I persist? (at least 10 times) • Daily variation, steady increase of portion size	• Tips about offering vegetables to children on a daily basis and the preparation of age appropriate vegetable meals	Mother feeding infant pureed vegetables/fruits	Five tips: Timing, routine, adequate pacing, stop at the right time, enjoy
3	8 m	Being creative with vegetables	• Increase level and variety of texture • Set a good example	• Additional information about introducing more lumpy foods to children. • Tips about preparing and storing age appropriate vegetable meals • Tips to cut costs	Child eating sandwich with mother; new topping on sandwich	What to do when infants a) want more autonomy during mealtimes and b) don’t want to eat
4	13 m	Vegetables are part of a balanced diet	• Eating with the whole family • Recommendations for vegetable intake	• Achieving the recommended intake for vegetables	Dinner with whole family; child is served a new vegetable	Positive ways of dealing with negative behavior during dinner
5	16 m	Keep eating vegetables	• Inform parents on possible food neophobia phase, and how to respond	• Involving children in the preparation of vegetables	Dinner with whole family; child is served something new	Inform parents on possible food neophobia phase, and how to respond to that

*Note.* m = months

The Stages of Change Model [83] is used to achieve behavior change. The model identifies five stages that people move through when modifying behavior; 1) pre-contemplation; 2) contemplation; 3) preparation; 4) action; 5) maintenance. During the first two sessions (during the 19 day feeding schedule) it is assumed that mothers are motivated to offer their child a vegetable puree daily (preparation/action phase). For session three to five, the stage of change is monitored based on the conversation with the mother. When the mother appears not to be motivated to offer vegetables or encounters barriers in doing so, the protocol contains a series of possible questions and arguments to be discussed to motivate or come up with solutions for the encountered barriers.

Interveners are explicitly not allowed to give advice on *how* to feed the infant to avoid overlap with the VIPP-FI intervention. If mothers have any specific questions about feeding issues, they are referred to their youth health care center or the website of the Dutch? Nutrition Centre where parents get standard advice available for the general public.

In summary, the standardized telephone protocol for each intervention session contains the following elements:

- General part with standardized questions about adherence of mother and child to the vegetable guidelines
- Classifying the stage of change
- Testing the extent to which goals (e.g. knowledge of the topics discussed) of the previous session were achieved by asking questions and repeating information when necessary (sessions 2, 3, 4, 5)
- Discussing the basic information material that mothers receive per post and presenting the option to tailor the conversation by addressing the optional information and questions the mother might have
- Discussing continuation and goal setting with regard to vegetable consumption (sessions 2, 3, 4, 5)

To optimize adherence of interveners to the intervention protocol, interveners familiarize themselves with all the information in the protocol and are trained on how to approach the mothers during the telephone calls. In addition, the interveners have regular meetings to discuss the RVE intervention, exchange experiences and discuss difficulties that may arise. To allow further monitoring of adherence and achievement of the intervention goals, notes are made of each interaction with the parent. In addition, important individual details and information discussed are noted.

## VIPP-feeding infants (VIPP-FI)

The VIPP-Feeding Infants intervention focuses on *how* to feed an infant. The intervention is based on an existing parenting intervention that has repeatedly been proven effective in enhancing both parental sensitivity in general and sensitive discipline in particular: the Video-feedback Intervention to promote Positive Parenting-Sensitive Discipline (VIPP-SD) [84]. For the present study, the VIPP-SD was adapted to the specific situation of feeding infants (VIPP-FI) and aims to enhance sensitive parenting during feeding. The intervention consists of five sessions that take place at home and makes use of a detailed protocol that can be requested from the first author, SV. To avoid overlap with the RVE intervention, interveners are explicitly not allowed to give any advice on what type of food to give the infant. If mothers have any specific questions about this, they are referred to their youth health care center or the Dutch Nutrition Centre.

The goal of VIPP-FI is to increase mothers’ sensitive reactions to her child’s hunger and satiety cues and to increase sensitive discipline and autonomy support during feeding. To reach this goal, mothers are shown videotapes of their own feeding-interaction with their infant and receive feedback on these tapes by a trained intervener. For each session a different type of meal-setting is filmed. The videos also include potentially challenging situations like introducing the child to a new taste. The mealtimes are filmed approximately one week before the session takes place, to allow the intervener to prepare the feedback they want to give mothers. The different settings that are filmed and topics that are discussed during each session are displayed in Table 4.

One of the core principles of VIPP is to always provide positive feedback to a mother [72]. Every moment where a mother shows sensitive ways of responding to infant cues of hunger, satiety, or other cues are pointed out during the sessions. Instances of insensitive behavior by the mother during the video are also discussed but the intervener always provides the mother with an alternative by referring to a more sensitive response that the mother showed during the video. In doing so, the mother becomes her own role model for showing sensitive reactions to the infant’s needs. Another core principle of VIPP is that to improve maternal sensitivity, mothers need to be trained in observing and interpreting the behavior of their child (in essence, how does my child signal hunger, satiety, interest in their surroundings, etc. [72]). Therefore, during the first sessions mothers do not get direct feedback on their own behavior, as this likely distracts them from observing the behavior of their infant while watching the video. In the standard VIPP protocol mothers do not get specific feedback on their own behavior until the third session. However, in VIPP-FI we allow interveners to do this from the second half of the second session. We made this alteration as there is a relatively long time gap between the second and third session (2 to 4 months) and we wanted to give mothers as many pointers as possible to practice sensitive feeding in the months between the sessions. Examples of techniques used for providing feedback to mothers are *speaking for the child* (i.e. the intervener stops the video and talks with a mother about what the infant is trying to communicate at that point in the video) and *corrective messages* (i.e. the intervener stops the video after an example of insensitive behavior of the mother and gives an example of a more sensitive approach she could have used and showed at another point during the video).

To ensure the adherence of interveners to the intervention protocol, interveners receive five days of training in VIPP-SD and a one-day training in VIPP-FI. Moreover, they perform the VIPP-FI in one pilot-family before performing the intervention for the present trial. The progress of the intervention in this pilot-family is discussed extensively with interveners who have experience with the VIPP-FI protocol. Adherence is further optimized by scheduling regular meetings with all interveners at each study location, where the progress of each family receiving the intervention is discussed, as well as any issues that may arise while providing the interventions. Finally, the interveners from both study sites have regular meetings to make sure that adherence is similar at both sites. Similar to the procedure in the RVE intervention, notes are made of each interaction with the parent to allow further monitoring of adherence and achievement of the intervention goals. In addition, important individual details and information discussed are noted.

## Vegetable exposure + VIPP-feeding infants (COMBI)

Participants randomly allocated to the combined intervention receive both the RVE intervention and the VIPP-FI as described above. Similar to these interventions, families receive five phone calls for the RVE intervention and five home visits for VIPP-FI, at the same moments as in the two separate interventions.

## Attention control condition (AC)

Participants in the attention control condition receive five phone calls, scheduled at the same time that the intervention sessions in the RVE, VIPP-FI and COMBI conditions take place. The researchers/students that make the phone calls are explicitly not allowed to give any advice on the what and how of complementary feeding; instead, they are instructed to simply inquire after the development of the child, using a semi-structured interview, listen to mothers and show interest and empathy. Topics that are discussed concern the general development of the child (e.g., sleeping behavior, motor development, language development) as well as what the mother’s experiences are with the complementary feeding of her child. If mothers have any specific questions about complementary feeding, they are referred to their youth health care center or the Dutch Nutrition Centre.

### Measures

#### Primary outcome measures

**Vegetable intake.** For the duration of the 19-day weaning schedule the child’s consumption of the purees is assessed. On days 1, 2, 18, and 19 of the feeding schedule researchers visit the families’ homes and measure the amount of the vegetables the infants eat in grams (maximum of 125 g per day, as this is the amount available per day). This is done by weighing the jar of food, bowl, spoon, bib and the cloth mother plans to clean the baby with both before and after the meal by using a standard small kitchen scale (Soehnle, Fiesta 65106). For the other days of the feeding schedule, mothers are asked to put all the leftover puree back in the jar as precisely as possible and store it in the fridge until the researchers collect the jars of food at day 18. The researchers determine the amount of puree eaten on these days by weighing the jars.

At *t*_*12*_, *t*_*18*_, *t*_*24*_, and *t*_*36*_ vegetable intake is measured by asking mothers to fill out web-based 24-h recalls on three randomly assigned, non-consecutive days using the online program, Compl-eat, developed by Wageningen University and Research. Compl-eat is based on the multiple pass method [85] to increase accuracy of dietary recalls and uses the Dutch food composition table [86] to calculate energy and nutrient intake. The program was adapted to assess the diets of infants and young children for this study (e.g., inclusion of smaller portion sizes, and special baby foods). The recall days are scheduled in advance. The parent is provided with a paper food diary to be filled out throughout the day if the child is not in the parents care, but for instance with a babysitter or at a day-care center, making it possible for the parent to enter the data in Compl-eat afterwards. In addition, the parent is asked to weigh all vegetables consumed by the child on a digital scale. Instructions on how to fill out Compl-eat are given during the home visits of *t*_*12*_, *t*_*18*_, *t*_*24*_, and *t*_*36*_; invitations to fill out the recalls are sent after the home visits.

**Vegetable liking** is measured every day of the feeding schedule by asking mothers to note their infants liking of the vegetables in a diary. Using the same scale as used in the trial by Barends and colleagues (2013), mothers are asked to rate their infant’s liking on a 9-point Likert scale, ranging from 1 (dislikes very much) to 9 (likes very much). At *t*_*12*_, *t*_*18*_, *t*_*24*_, and *t*_*36*_, liking of the target and control vegetables (cauliflower and green beans) is measured using the same scale, filled out by the mother.

Child **self-regulation of energy-intake** is measured using questionnaires and observation. Mothers are asked to fill out the Baby Eating Behavior Questionnaire (BEBQ [87]) at *t*_*0*_ and the Child Eating Behavior Questionnaire – Toddler (CEBQ-T [88]) at all other *t*’s. The BEBQ and CEBQ-T assess several aspects of eating behavior including satiety responsiveness and food responsiveness. These scales are used as indicators of the infant’s self-regulation of energy-intake.

In addition, at *t*_*18*_, *t*_*24*_ and *t*_*36*_, a home-based *eating in the absence of hunger (EAH)* paradigm is used. This is done according to the free-access procedure, which is considered the gold-standard for this type of measurement [89–92]. During the home visit the researcher carefully assesses what and how much the child eats during dinner to determine the weight, energy and macronutrient content of the meal. In addition, the mother is asked to indicate how satiated she thinks her child is after consuming dinner. Directly after dinner an 8-min free play session takes place after which the researcher provides a plate with savory and sweet age-appropriate snacks and the child is told that these are for him/her to eat. The mother is asked not to interfere with the child’s behavior during this time. Using these data, the EAH-score, the percentage of energy intake from the snacks relative to the energy intake from the dinner, is calculated.

#### Secondary outcome measures

**Child anthropometrics** are measured at all *t*’s. Infants’ body weight is measured by asking mothers to first stand on a calibrated electronic personal scale (KERN MPC/SECA robusta 813) themselves, and then again while holding their infant. The difference between these two weights produces the child’s weight. As of *t*_*24*_, children are invited to stand on the scales themselves. Weight is measured in 0.1 kg. Infants’ length is measured by lying them down on a small mat with an indication of centimeters printed on top of it. As of *t*_*24*_ child length is measured using a stadiometer (SECA 213, Chino, USA/Garant).

**Child eating behavior** is measured by the mother-reported Baby Eating Behavior Questionnaire at *t*_*0*_ (BEBQ [87]) and the Child Eating Behavior Questionnaire – Toddler (CEBQ-T [88]) at all other *t*’s. The BEBQ and CEBQ-T are both derived from the Child Eating Behavior Questionnaire (CEBQ), a well-validated, reliable and widely used questionnaire that assesses different aspects of child eating behavior [93, 94]. We use the CEBQ-T as of *t*_*1*_ as it is more appropriate for assessing children’s eating behavior in relation to eating solid foods. However, since the scale ‘emotional over-eating’ is largely inapplicable for infants under the age of 2 years (e.g., “My child eats more when upset”) this scale is only added to *t*_*18*_*, t*_*24*_ and *t*_*36*_*.*

**Maternal feeding behavior** is measured using both observations of family meals at home and questionnaires. When the child is 4–7 months of age (*t*_*0*_ and *t*_*1*_), a videotape is made of the mother feeding the child one of the pureed foods of the feeding schedule. At all other time points, a family dinner is videotaped. These videos are coded by trained researchers/students for maternal sensitive feeding using the Ainsworth scale [95]. In addition, maternal responsiveness to child satiety cues is coded using a scale based on the Responsiveness to Child Feeding Cues Scale [96], and maternal pressure to eat is coded using a scale based on a large Dutch study that observed family meals in 4–6 year-olds [66].

In addition, at each time point the Infant Feeding Style Questionnaire [97] is administered. This questionnaire has shown adequate internal consistency and validity and measures the following parental feeding styles: laissez-faire, restrictive, pressuring, responsive and indulgent. As of *t*_*18*_ the following scales from the validated Comprehensive Feeding Practices Questionnaire [98, 99] are added which are appropriate at that age: restriction, monitoring, modelling, encourage balance and variety, pressure to eat, child control, emotion regulation and food as reward. Scales from the Feeding Practices and Structures Questionnaire [100] are also added as of *t*_*18*_ (reward for eating, overt restriction) and *t*_*24*_ (reward for behavior, persuasive feeding, structured meal setting, structured meal timing).

#### Other measures

The following potential covariates will be assessed: demographic variables such as maternal and paternal education and job status, family income, cultural background (*t*_*0*_); type of milk feeding (breast/formula: *t*_*0*_*-t*_*18*_); maternal depression (*t*_*0*_*-t*_*36*_: Center for Epidemiologic Studies Depression Scale [101]); maternal vegetable intake (*t*_*12*_ and *t*_*36*_: Food frequency questionnaire [102]); maternal anthropometrics (*t*_*0*_*-t*_*36*_); use and amount of purée consumed of the 100 distributed vegetable- and fruit jars in the 5 months after the feeding schedule (*t*_*12*_); maternal self-efficacy related to feeding their child (*t*_*0*_*-t*_*36*_: Parental Feeding Self-Efficacy Questionnaire [103]); maternal emotions during feeding the child (*t*_*0*_*-t*_*36*_: measure designed for this study); structure of family meals (*t*_*0*_*-t*_*36*_: Meals in our Household [104]); maternal perception of feeding (*t*_*0*_*-t*_*36*_: Five Minute Speech Sample [105]); child temperament (*t*_*0*_*-t*_*12*_: Infant Behavior Questionnaire-Revised [106]; *t*_*18*_*-t*_*36*_: Early Childhood Behavior Questionnaire [107]); general parenting styles (*t*_*0*_*-t*_*36*_: observed maternal intrusiveness during mealtimes and observed maternal sensitivity and intrusiveness during free-play situations [95]; *t*_*18*_*-t*_*36*_: Comprehensive General Parenting Questionnaire [108]).

### Blinding

Researchers coding video data are blinded for intervention-allocation. It is impossible to blind participants for intervention-allocation, because they will be informed prior to randomization about what types of advice they can receive in the study and it will be clear after randomization what type of advice they are receiving.

### Participant reimbursement and efforts to prevent drop-out

As a compensation for the time and effort participants invest in our study, families receive several compensations. Apart from the pureed vegetables or fruits during the feeding schedule and the 100 jars of baby foods until the infant is 12 months of age, families receive gift tokens of 25 euros and a gift for the child of approximately 5 euros at *t*_*18*_*, t*_*24*_*,* and *t*_*36*_. Additionally, all videos made throughout the study are shared with the families at completion of the study, and families randomly allocated to receive VIPP-FI receive the videos used for the intervention during the last session of the intervention.

To involve participants in the study we will send families biannual newsletters about the study, mentioning interesting facts (e.g., inclusion rates, presentations at symposia, pictures of researchers/students involved in the project). Also, we aim to stimulate a pleasant relationship between researchers and participating mothers by for example sending birthday cards to the family when the child will have its birthday. In a similar effort, and to diminish any additional burden for the participating families, we will strive to provide continuity in the researchers/students that are in direct contact with a family (e.g., at home visits or telephone calls). Moreover, we will make sure during every home visit to check whether participants have any questions about the measurements and/or interventions and to provide assistance in filling out questionnaires or dietary recalls whenever needed.

### Confidentiality, data management and access

All data will be stored using numbers to identify participants at the secured databases of Leiden University and Wageningen University and Research. Only one document exists that links participant numbers to personal data, and this file is only available to the main researchers performing data collection at Leiden University and Wageningen University. Data that need to be entered manually (e.g., measured weight and height during home visits, codes of video material) will be entered in the latest version of the statistical software package IBM SPSS Statistics by trained researchers/students. The quality of this data entry will be checked regularly by another (independent) trained researcher/student.

As detailed in the consortium agreement-contract of the project, only researchers and students involved in the project working at any of the academic parties (Leiden University, Wageningen University and Research) will be allowed access to the data. With the exception of the video-recordings (VIPP-FI), which contain privacy-sensitive information, research data will be open access where possible (e.g. when a peer-reviewed journal requests or offers the uploading of anonymized datasets into an open access database. In these cases, all personal information will be removed from data files and replaced by participant identification numbers. The file linking these numbers to personal information will be stored digitally in a separate password protected file that will only be accessible to the researchers). Large video-files will be shared between the two universities by making copies on external pass-word protected hard-drives and personally exchanging these hard-drives.

### Analyses

The intention-to-treat principle will be applied to all analyses. Whether the interventions differentially affect primary and secondary outcomes over time will be analyzed using linear mixed models analyses, a technique that makes use of every data point for every participant, irrespective of their missing data. The three intervention groups will be compared to the control group, and the combined group will be compared to the repeated exposure and the VIPP-FI group. A significance level of α = .05 will be used. The analyses will be corrected for relevant covariates such as family socioeconomic status, maternal consumption of vegetables, parental body mass index (BMI), child temperament, etc.

### Monitoring of interventions and trial progression

Participants will be asked to fill out an evaluation form concerning the interventions following the last session. These forms will assess participants’ satisfaction with the intervention as well as with the person delivering the intervention. In addition, participants will be asked to note any other comments about the interventions, allowing for spontaneously reported adverse events. As the interventions are not invasive and merely provide parents support, advice and commercially available foods with a history of safe use, no adverse events are expected and no stopping guidelines are formulated. For the same reasons, a data management committee is not needed. Principle investigators at each study site (i.e. JM, SV, KG, JV and GJ) will supervise data collection and data management. We will not perform any interim analyses as we want to avoid the risk of the results of such analyses influencing the overall results of the trial. No explicit trial conduct audit is planned; however, yearly reports on the progress of the project will be sent to the major funder of the trial (The Netherlands Organization for Scientific Research). If any major changes will occur in the study protocol (e.g., changes to outcomes or assessment periods) the ethical review boards that approved the study as well as the funder of the trial will be notified of these changes.

### Dissemination policy

It is planned to publish the results of our trial in peer-reviewed journals, as well as present the results at (inter) national conferences. Also, participants will receive a report of the results of our study after completion of the study. Publication in magazines for healthcare professionals and the general public are also intended. Authorship to any publications will be granted to those who fulfil the ICMJE recommendations [109]. We will not hire any professional writers.

## Discussion

*Baby’s First Bites* will be the first trial explicitly testing the separate and combined effects of promoting the *what* and *how* of complementary feeding. By comparing three prolonged, intensive interventions, we will be able to draw firm conclusions on what is most important to focus on when promoting vegetable acceptance and children’s self-regulation of energy intake in early childhood; what food to offer, how to offer this food, or a combination of the two. Moreover, this will be the first trial to include an intervention specifically manipulating sensitive feeding practices without manipulating any other variables, evaluating its effects using both self-report and observational measures. This allows conclusions on whether this parenting practice will indeed promote healthier food preferences in children and will foster children’s ability to self-regulate their energy intake, as is often suggested in the literature.

The planned study also provides some points of discussion to be considered. First, the channels of recruitment we have chosen pose the risk that participating families are not representative of the general population, as they are partly recruited from a database of pregnant women who showed interest in information about infant nutrition. Thus, these families may be more motivated to provide a healthy eating environment for their infant than the general public. However, it should be noted that time-consuming randomized controlled trials (RCTs) like the present study will always elicit this potential selection bias, irrespective of the channels of recruitment chosen. Also, this drawback is negated somewhat by the fact that this study succeeded in including participants at all educational levels. Nevertheless, this potential selection bias should be taken into account when considering the implementation of the results of this study. Second, we chose to give parents the opportunity to start complementary feeding from the age of 4 months, thereby making sure that we followed parental preferences in starting complementary feeding. There is still some discussion in the literature about when to start complementary feeding. The general recommendation from the World Health Organization (WHO) is to exclusively breastfeed until the age of 6 months and introduce complementary foods from 6 months [1]. For the European Region, WHO [110] recommend that all infants should be exclusively breastfed from birth to about 6 months of age, and at least for the first 4 months of life, but that some infants may need complementary foods before 6 months of age, and that these should not be introduced before 4 months. The European Food and Safety Authority (EFSA) panel [111], the European Society for Paediatric Gastroenterology, Hepatology and Nutrition (ESPGHAN) [112] recommend that complementary foods including allergens are introduced between 4 and 6 months, and this has been shown to be associated with a reduced risk of food allergies [113]. Starting complementary feeding between 4 and 6 months is also in accordance with recommendations from the Dutch Nutrition Centre [114] and the Dutch youth health care centers [115] and thus reflects official Dutch guidelines and probably the daily practice of parents in the Netherlands.

Third, we chose to deliver the combined intervention by simply following the same procedures as used in each separate intervention, and the intervention was provided by two different researchers/students (one delivering RVE, and one delivering VIPP-FI). As such, it can be debated whether this really constitutes a *combined* intervention or simply *two* interventions. Also, from the families’ point of view, receiving advice from two different persons might not be ideal. An alternative approach would have been to incorporate all information of both interventions in the home visits. However, we decided against this as the VIPP-FI home visits already took up 60 to 90 min. Including the information of the RVE intervention in this session would result in too much information for the mother to properly process in one sitting, increasing the risk that the effects of the intervention would diminish. Fourth, considering the time-consuming nature of this study for families, there will be a considerable risk of drop-out during the study. This risk is even higher in the selected sample of first-time mothers, as it is likely that many families will expand their family during the study period, making the time they have available for participating in this study more limited. We plan to accommodate families as much as possible to make sure that they will be able to finish the study, for instance by offering assistance where necessary (e.g., filling out questionnaires together or sending personal reminders) and by being flexible in planning the home-visits.

Finally, if the proposed RCT will prove the interventions effective, the labor intensiveness of the tested interventions may pose problems for their implementation to the general public. Although this is not so much a limitation of the current study, it is a drawback for implementing its results, as it will be necessary to translate the interventions to scalable prevention programs before the interventions can be implemented for a larger group.

In conclusion, the planned trial has the potential to provide valid evidence on the question how parents may promote healthy eating habits from the very first start of eating solid foods. If proven effective, these interventions could be useful to large scale effective prevention of childhood obesity.

## Data Availability

The anonymized datasets that will be analyzed during the current study will be made open access where possible. Video files are not publicly available for privacy reasons.

## Abbreviations

AC: attention control group
BEBQ: Baby Eating Behavior Questionnaire
BFB: Baby’s First Bites
BMI: Body Mass Index
CEBQ: Child Eating Behavior Questionnaire
CEBQ-T: Child Eating Behavior Questionnaire-Toddler
COMBI: combined RVE + VIPP-FI
EAH: eating in the absence of hunger
EFSA: European Food and Safety Authority
ESPGHAN: European Society for Paediatric Gastroenterology, Hepatology and Nutrition
IM: Intervention Mapping
MI: Motivational Interviewing
RCT: Randomized Controlled Trial
RVE: repeated vegetable exposure
VIPP-FI: Video-feedback Intervention to promote Positive Parenting-Feeding Infants
VIPP-SD: Video-feedback Intervention to promote Positive Parenting-Sensitive Discipline
WHO: World Health Organization
